# Antipsychotic polypharmacy in a regional health service: a population-based study

**DOI:** 10.1186/1471-244X-12-42

**Published:** 2012-05-15

**Authors:** Miguel Bernardo, Anna Coma, Cristina Ibáñez, Corinne Zara, Josep Maria Bari, Antoni Serrano-Blanco

**Affiliations:** 1 Schizophrenia Clinic Program, Department of Psychiatry, Neuroscience Institute, Hospital Clinic de Barcelona, Barcelona, Spain; 2 Department of Psychiatry and Clinical Psychobiology, Health Sciences Division, University of Barcelona, Barcelona, Spain; 3 Institut d’Investigacions Biomèdiques August Pi i Sunyer (IDIBAPS), Barcelona, Spain; 4 Centro de Investigación Biomédica en Red de Salud Mental (CIBERSAM), Barcelona, Spain; 5 Pharmacy Direction. Catalan Health Service, Barcelona, Spain; 6 Parc Sanitari Sant Joan de Déu, Servicios de Salud Mental y Fundación Sant Joan de Déu, Red de Investigación en Actividades Preventivas y Promoción de la Salud (RedIAPP), Barcelona, Spain

**Keywords:** Antipsychotics, Clozapine, Antipsychotic combination, Outpatient setting

## Abstract

**Background:**

To analyse the extent and profile of outpatient regular dispensation of antipsychotics, both in combination and monotherapy, in the Barcelona Health Region (Spain), focusing on the use of clozapine and long-acting injections (LAI).

**Methods:**

Antipsychotic drugs dispensed for people older than 18 and processed by the Catalan Health Service during 2007 were retrospectively reviewed. First and second generation antipsychotic drugs (FGA and SGA) from the Anatomical Therapeutic Chemical classification (ATC) code N05A (except lithium) were included. A patient selection algorithm was designed to identify prescriptions regularly dispensed. Variables included were age, gender, antipsychotic type, route of administration and number of packages dispensed.

**Results:**

A total of 117,811 patients were given any antipsychotic, of whom 71,004 regularly received such drugs. Among the latter, 9,855 (13.9%) corresponded to an antipsychotic combination, 47,386 (66.7%) to monotherapy and 13,763 (19.4%) to unspecified combinations. Of the patients given antipsychotics in association, 58% were men. Olanzapine (37.1%) and oral risperidone (36.4%) were the most common dispensations. Analysis of the patients dispensed two antipsychotics (57.8%) revealed 198 different combinations, the most frequent being the association of FGA and SGA (62.0%). Clozapine was dispensed to 2.3% of patients. Of those who were receiving antipsychotics in combination, 6.6% were given clozapine, being clozapine plus amisulpride the most frequent association (22.8%). A total of 3.800 patients (5.4%) were given LAI antipsychotics, and 2.662 of these (70.1%) were in combination. Risperidone was the most widely used LAI.

**Conclusions:**

The scant evidence available regarding the efficacy of combining different antipsychotics contrasts with the high number and variety of combinations prescribed to outpatients, as well as with the limited use of clozapine.

## Background

Antipsychotic drugs are often combined in the clinical treatment of schizophrenia, despite the lack of a solid evidence base for this strategy. Indeed, the combination of antipsychotics has yet to be studied in sufficient detail, although numerous pharmaco-epidemiological studies describing the phenomenon of antipsychotic polypharmacy have been conducted internationally. However, these studies are highly heterogeneous and differ in terms of methodology, sample size, chronology, diagnostic criteria, the treatments included and the salvage therapy options [1-8].

The prevalence of antipsychotic polypharmacy in the USA ranges from 7% to around 50% [9-18], including a study in child and adolescent population [19], with most studies reporting rates between 10 and 30%. Similarly, European data on patients diagnosed with schizophrenia and being treated in acute psychiatric units reveal that antipsychotic polypharmacy is a common (if not the most common) strategy and that it is not reserved for the most treatment-resistant cases [20,21].

Some authors have also reported a trend towards an increased use of antipsychotic polypharmacy in the same patient over time [11,22], although evidence-based guidelines only recommend concomitant antipsychotic treatment after several failed attempts at monotherapy, including with clozapine [23,24]. Indeed, the various clinical guidelines with stronger consensus [25-27] place clear limitations on the use of antipsychotic polypharmacy.

It has been suggested that some antipsychotics, both first (FGA) and second (SGA) generation, are especially used in polytherapy, whereas others, such as clozapine, tend to be used as monotherapy [28]. This contrasts with the large number of published studies on the use of clozapine in combination as opposed to many of the combinations that are currently used [29,30]. There is no specific antipsychotic combination recommended in the current guidelines [25-27]. In this regard, it would also seem advisable to analyse the use of long-acting injections (LAI) of antipsychotics and determine their profile of use in monotherapy or combination.

In the case of schizophrenia, resistance to treatment and the unsatisfactory functional outcomes remain a significant problem in both the clinical context and in terms of public health [9,31-33]. To date, clozapine is the only treatment to have shown significantly better outcomes than other antipsychotics in partial or non-responder patients to antipsychotic monotherapy [34-36]. Furthermore, although numerous maximization strategies have been tried in randomised controlled studies, none of them has proved the effectiveness in non-responders to monotherapy [1-4]. On the other hand, in some cases antipsychotics could be associated to treat some symptoms like insomnia, especially clotiapine.

As regards the safety of antipsychotic polypharmacy, there are data which suggest that its use does not increase side effects or the risk of death by natural causes [37], although some studies do report a moderate and additive risk of adverse effects [38].

The present study sought to evaluate the profile of antipsychotic drugs dispensation in a broad sample over a one-year period (2007). The aim was to determine the extent to which these dispensations were in line with current guidelines and to indicate to policy makers, managers, clinicians and trainee physicians those areas which require further research.

## Methods

### Setting

The study was conducted in the Barcelona Health Region (BHR), which provides healthcare to 5,105,555 people, making it the largest in Catalonia. Catalonia is an autonomous region in the northeast of Spain that covers an area of 32,114 km², its official population in December 2007 being 7,503,118 inhabitants. Medical consultations and hospital admissions are fully covered by the local Catalan Health Service (CatSalut). Prescriptions are free to retired people, while their cost is partially covered for those in employment. In the BHR, 81.0% of people entitled to public healthcare are older than 18, and of these, 51.6% are women (data from the local central register for December 2007).

### Study population

Using the database of official medical prescriptions (DATAMART®) processed by the Catalan Health Service we selected all out-patient prescriptions for one or more antipsychotic drugs prescribed by a physician of CatSalut (independently of the speciality) to a person over the age of 18 in the BHR, and dispensed during 2007 in a pharmacy in Catalonia. Antipsychotic drugs prescribed to inpatients at hospital units were not considered for the purposes of the present study.

The antipsychotic drugs analysed were all those included (with the exception of lithium) under the code N05A of the Anatomical Therapeutic Chemical (ATC) classification system and marketed in Spain. FGAs (chlorpromazine, clotiapine, fluphenazine, haloperidol, levomepromazine, perphenazine, periciazine, pimozide, pipothiazine, sulpiride, tiapride, trifluoperazine and zuclopenthixol) were distinguished from SGAs (amisulpride, aripiprazole, clozapine, olanzapine, quetiapine, risperidone, sertindol and ziprasidone), as well as oral formulations from long-acting injections (LAI) (fluphenazine LAI, pipothiazine LAI, risperidone oral and LAI, and zuclopenthixol oral and LAI).

### Outcome

The aim of the study was to apply a retrospective epidemiological analysis to determine the use of antipsychotic treatments in combination and as monotherapy, with particular attention to the use of clozapine and injectable forms.

**Figure 1:**
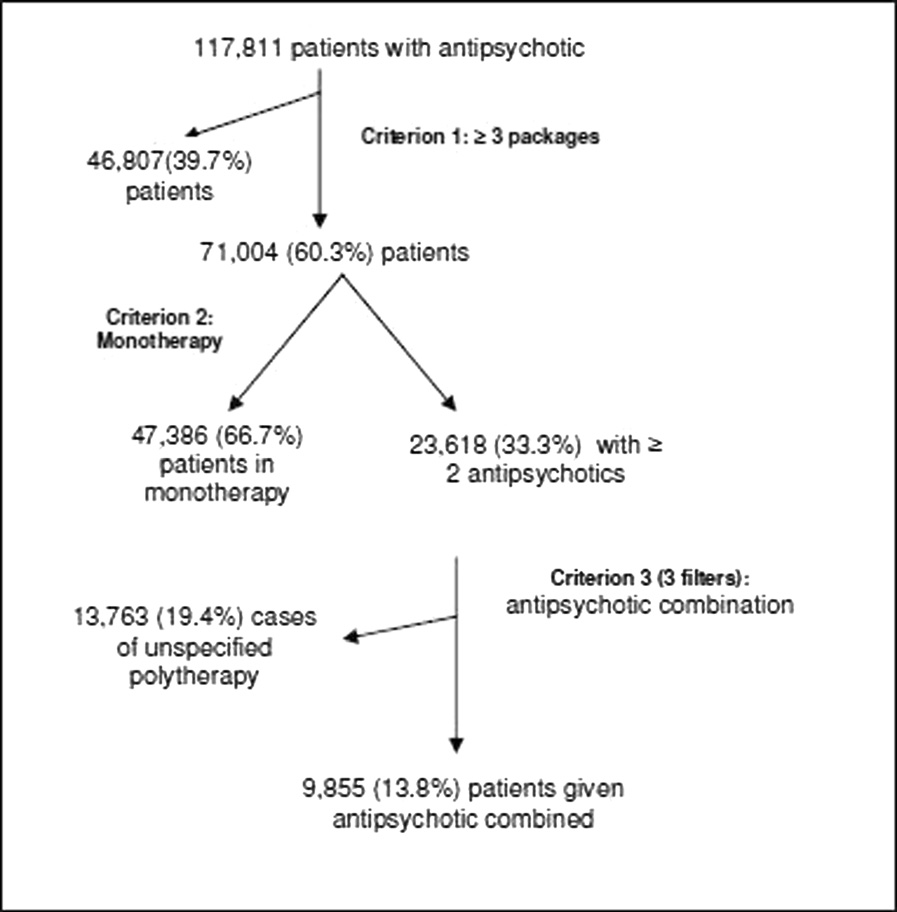
Selection algorithm for patients with antipsychotics.

Three criteria were established to define more specifically the study sample (Figure 1).

Criterion 1: **Regularly-dispensed antipsychotic treatment**: at least three packages of any antipsychotic dispensed during 2007.

Criterion 2: **Monotherapy**: for those patients who were given at least three packages of an antipsychotic a distinction was made between those prescribed just one antipsychotic (monotherapy) and those who received two or more different antipsychotics (antipsychotic **polytherapy**).

Criterion 3: Antipsychotic **combination treatment**: for those patients receiving antipsychotic polytherapy, further three filters were applied, based on a review of the literature [34-36], to define the criterion of antipsychotic combination:

1- At least two packages of two or more antipsychotic drugs dispensed in the three months prior to the last prescription supplied.

2- The filter 1 was not fulfilled, but more than one package was dispensed in the fourth month prior to the last prescription supplied.

3- Filters 1 and 2 were not fulfilled, but at least six packages of each antipsychotic were dispensed during the year in question.

We assumed that, in general, at least 1 box of medication covers 1 month of treatment. Further on, we developed different filters to precise the identification of patients with antipsychotic combinations. Criterion 1 was used to identify the population with regular dispensation of antipsychotics. Criterion 2 was used to distinguish patients who received one antipsychotic from those who received more than one. In any case, receiving more than one during a year does not mean that they were used simultaneously; consequently we applied the 3rd criterion to identify the simultaneous use of antipsychotics and distinguish it from the sequential use.

In the 3rd criterion, Filter 2 is used to detect patients to whom the 1st filter does not apply because multiple packages have been dispensed simultaneously during the 4th month prior to the last dispensation and consequently the patient has enough medication to cover a longer period.

The variables recorded referred to demographics (age and sex) and prescription characteristics (antipsychotic, route of administration and packages dispensed). No personal or clinical data of patients have been collected. This study has been realized with general data obtained from the pharmacy prescription database, not with individual data of subjects. Therefore, no ethical approval had to be obtained for this study.

### Data analysis

A classification algorithm, implemented in Visual Basic for Applications (VBA), was designed to classify those patients who met the criteria for receiving antipsychotic drugs in combination. The results of the tables are obtained from descriptive statistics. The comparisons between proportions were made using a Z-test with a level of significance of 0.05. This statistical analysis was performed using *SPSS v. 16.0*.

## Results

The initial analysis identified 117,811 patients who had been dispensed at least one antipsychotic during 2007. Application of the above mentioned selection criteria revealed that of these, 71,004 had been regularly dispensed an antipsychotic drug, of whom 47,386 (66.7%) were given the antipsychotic as monotherapy and 9,855 (13.9%) received more than one antipsychotic combined. The remaining 13,763 (19.4%) were classified as cases of unspecified antipsychotic polytherapy as they did not fulfil criterion number 3 (Figure 1).

**Figure 2:**
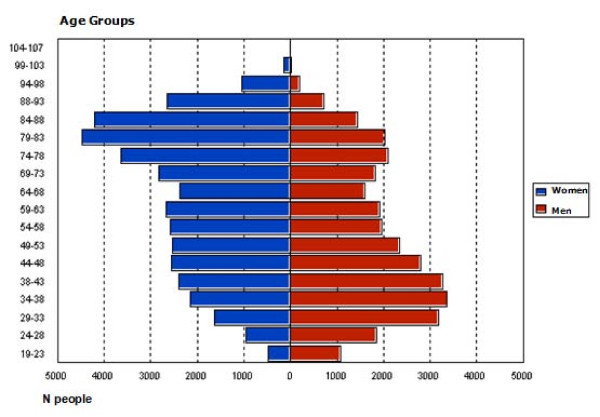
Distribution of the 71,004 patients who are given antipsychotics according to age and sex.

Of the 71,004 people who were regularly dispensed antipsychotic medication, 55.4% were women and 52.2% were aged between 19 and 60 years (Table 1). Figure 2 shows the sample distribution according to age and sex. It can be seen that the use of antipsychotics follows a bimodal curve, the biggest users being men younger than 54 and women older than 73.

**Table 1:** Distribution of the study sample according to age and sex

**Age (years)**	**Initial Sample (N = 71,004)**			**Antipsychotic monotherapy (n = 47,386)**	**Antipsychotics combined (n = 9,855)**	
	**Women N(%)**	**Men N(%)**	**Women ** **N(%)**	**Men N(%)**	**Women N(%)**	**Men ** **N(%)**
19-40	6,144 (15.6)	10,808 (34.1)	3,504 (12.6)	5,908 (30.1)	1,153 (27.8)	2,517 (44.1)
41-60	10,242 (26.0)	9,843 (31.1)	6,589 (23.7)	5,697 (29.0)	1,560 (37.7)	2,297 (40.2)
61-80	12,108 (30.8)	7,455 (23.5)	9,277 (33.4)	5,421 (27.6)	900 (21.7)	727 (12.7)
>80	10,824 (27.5)	3,580 (11.3)	8,379 (30.2)	2,611 (13.3)	529 (12.8)	172 (3.0)
Total	39,318 (55.4)	31,686 (44.6)	27,749 (58.6)	19,637 (41.4)	4,142 (42.0)	5,713 (58.0)

The most widely dispensed drugs were oral risperidone (30.3%), olanzapine (25.2%), quetiapine (18.7%) and haloperidol (15.6%) (Table 2, only shown those drugs that were used by more than 1% of the total sample).

**Table 2:** Distribution of the population according to the antipsychotic used (the table only shows those drugs that were used in more than 1% of the total sample)

**Drug**	**Initial sample (N = 71,004)**		**People given monotherapy** **(N = 47,386)**	**People given antipsychotics in combination** **(N = 9,855)**
	**People N(%)**	**packages N(%)**	**N(%)**	N(%)(*)
Risperidone (oral)	21,537 (30.3)	137,234 (13.8)	11,589 (24.5)	3,586 (36.4)
Olanzapine	17,895 (25.2)	174,715 (17.6)	9,619 (20.3)	3,657 (37.1)
Quetiapine	13,292 (18.7)	154,408 (15.5)	5,913 (12.5)	2,883 (29.3)
Haloperidol	11,053 (15.6)	91,328 (9.2)	4,431 (9.4)	2,750 (27.9)
Sulpiride	8,274 (11.7)	75,183 (7.6)	6,933 (14.6)	360 (3.7)
Levomepromazine	7,503 (10.6)	86,794 (8.7)	2,435 (5.1)	2,607 (26.5)
Clothiapine	4,416 (6.2)	33,490 (3.4)	628 (1.3)	2,157 (21.9)
Risperidone (LAI)	3,146 (4.4)	60,524 (6.1)	654 (1.4)	1,500 (15.2)
Amisulpride	2,910 (4.1)	24,957 (2.5)	655 (1.4)	1,253 (12.7)
Aripiprazole	2,855 (4.0)	23,551 (2.4)	664 (1.4)	1,025 (10.4)
Ziprasidone	2,560 (3.6)	19,757 (2.0)	721 (1.5)	875 (8.9)
Perphenazine	2,146 (3.0)	15,616 (1.6)	797 (1.7)	623 (6.3)
Clozapine	1,615 (2.3)	35,222 (3.5)	667 (1.4)	652 (6.6)
Fluphenazine	1,403 (2.0)	6,113 (0.6)	212 (0.4)	531 (5.4)
Chlorpromazine	1,209 (1.7)	9,577 (1.0)	369 (0.8)	387 (3.9)
Zuclopenthixol (LAI)	1,175 (1.7)	15,406 (1.5)	202 (0.4)	659 (6.7)
Trifluoperazine	1,163 (1.6)	11,912 (1.2)	280 (0.6)	367 (3.7)

*LAI* long-acting injection.

(*) Percentage with respect to the total number of patients given antipsychotics in combination (9,855).

### Antipsychotics in combination

Application of the three filter criterion to patients prescribed two or more antipsychotic drugs during the year in question revealed that 9,855 people were given a combination of antipsychotics. The age and sex distribution of these patients are shown in Table 1. Men accounted for 58.0% of those patients, more frequently in the age range 19–40. Conversely, in women antipsychotic polypharmacy was more frequent in the age range 41–60. Overall, however, antipsychotic combination was most likely prescribed to both men and women aged 19–60 (84.3% and 65.5%, respectively).

The frequency of drug combinations is displayed in Table 2. The antipsychotics that showed the highest rates of use in polytherapy were olanzapine (37.1%), oral risperidone (36.4%), quetiapine (29.3%), haloperidol (27.9%), levomepromazine (26.5%) and clotiapine (21.9%).

The combination of two antipsychotics was present in 5,692 patients (57.8%), in a total of 198 different combinations. The most common association was oral risperidone plus quetiapine (6.2% of the total number of associations; Table 3). Combinations of three antipsychotics were present in 2,519 patients (25.6%), with a total of 526 different combinations. Finally, 1,029 people (10.4%) received four antipsychotics in combination, while 615 patients (6.2%) were prescribed five or more.

**Table 3:** Combinations of two antipsychotics (the table only shows those combinations used in more than 2% of patients)

**Antipsychotics**	**Count**	**Percent**
Risperidone (oral) + Quetiapine	355	6.2
Risperidone (oral) + Haloperidol	272	4.8
Haloperidol + Levomepromazine	266	4.7
Risperidone (oral) + Olanzapine	237	4.2
Risperidone (oral) + Levomepromazine	232	4.1
Olanzapine + Haloperidol	226	4.0
Olanzapine + Quetiapine	216	3.8
Haloperidol + Quetiapine	204	3.6
Olanzapine + Levomepromazine	189	3.3
Olanzapine + Clothiapine	176	3.1
Risperidone (oral) + Risperidone LAI	162	2.9
Olanzapine + Amisulpride	131	2.3
Olanzapine + Risperidone LAI	123	2.2
Levomepromazine + Quetiapine	118	2.1
Risperidone (oral) + Clothiapine	115	2.0

*LAI* long-acting injection.

As regards the total number of combinations according to the type of antipsychotic, 6,106 (62.0%) of patients were dispensed both FGAs and SGAs, while 2,699 (27.4%) received different combinations of SGAs and 1,050 (10.6%) used different combinations of FGAs.

With respect to the route of administration, most combinations referred to oral antipsychotics (7,193 patients, 73.0%). Table 4 shows the data for combined oral and LAI treatment, which was present in 2,649 (26.9%) of patients given more than one antipsychotic. The most common combination was a second generation LAI with an oral SGA (26.2%). Finally, thirteen people were given a combination of different LAI antipsychotics.

**Table 4:** Patients with antipsychotic combinations according to type and route of administration

		**ORAL**		
		**FGA**	**SGA**	**FGA and SGA**
LAI	FGA	236 (8.9%)	420 (15.8%)	505 (19.1%)
	SGA	135 (5.1%)	693 (26.2%)	507 (19.1%)
	FGA and SGA	6 (0.2%)	63 (2.4%)	84 (3.2%)

*LAI *long-acting injection.

*FGA *first generation antipsychotic.

*SGA *second generation antipsychotic.

### Antipsychotics in monotherapy

Women accounted for 58.6% of people in antipsychotic monotherapy. Table 1 shows that men aged 19–40 were dispensed more antipsychotic drugs than their female counterparts (30.1% versus 12.6%), whereas women accounted for a higher proportion of dispensations in those aged over 80 (30.2% vs. 13.3%).

A total of 81.3% of people were given monotherapy with one of the following five drugs: oral risperidone (24.5%), olanzapine (20.3%), sulpiride (14.6%), quetiapine (12.5%) or haloperidol (9.4%) (Table 2).

### Use of clozapine

Clozapine was dispensed to 1,615 patients (2.3%) in the study sample (N = 71,004), of whom 65.4% were men. Of those patients on antipsychotic monotherapy, 667 (1.4%) were given clozapine. In combination therapy, 652 (6.6%) patients were dispensed clozapine, 345 of them in combinations of three or more antipsychotics.

Table 5 shows the different combinations of two antipsychotics that included clozapine (n = 307), the most frequent being clozapine plus amisulpride (22.8%).

**Table 5:** Patients with two antipsychotics combined including clozapine

	**Antipsychotics**	**Count**	**Percent**
Clozapine	Amisulpride	70	22.8
Clozapine	Haloperidol	35	11.4
Clozapine	Risperidone (oral)	31	10.1
Clozapine	Aripiprazole	29	9.4
Clozapine	Ziprasidone	27	8.8
Clozapine	Quetiapine	20	6.5
Clozapine	Levomepromazine	19	6.2
Clozapine	Zuclopenthixol (LAI)	14	4.6
Clozapine	Clothiapine	13	4.2
Clozapine	Olanzapine	13	4.2
Clozapine	Perphenazine	11	3.6
Clozapine	Risperidone (LAI)	11	3.6
Clozapine	Sulpiride	4	1.3
Clozapine	Pimozide	3	1.0
Clozapine	Chlorpromazine	2	0.7
Clozapine	Pipothiazine	2	0.7
Clozapine	Fluphenazine	1	0.3
Clozapine	Trifluoperazine	1	0.3
Clozapine	Zuclopenthixol (oral)	1	0.3
Total		307	100

*LAI *long-acting injection.

### Use of long-acting injections of antipsychotics

Of the study sample, 3,800 (5.4%) of patients were given a LAI antipsychotic. LAIs were dispensed to 2.4% of patients who received antipsychotic monotherapy and to 27.0% of those who were given combinations. Risperidone was the most commonly used LAI, both in monotherapy and in polytherapy (Table 6).

**Table 6:** Type of antipsychotic combinations in patients with LAI antipsychotics

	**Monotherapy** **(N = 47,386)**	**Combination with one oral antipsychotics (N = 5,692)**		**Combination with ≥2 antipsychotics(N = 4,163)**
		**FGA**	**SGA**	
Risperidone LAI	654 (1.4%)	95 (1.6%)	445 (7.8%)	948 (22.7%)
Fluphenazine	212 (0.4%)	49 (0.8%)	74 (1.3%)	405 (9.7%)
Zuclopenthixol LAI	202 (0.4%)	46 (0.8%)	178 (3.1%)	424 (10.1%)
Pipothiazine	70 (0.1%)	27 (0.4%)	49 (0.8%)	111 (2.6%)
TOTAL LAI	1,138 (2.4%)	217 (3.8%)	746 (13.1%)	1,699 (40.8%)

*LAI *long-acting injection.

*FGA *first generation antipsychotic.

*SGA *second generation antipsychotic.

## Discussion

This study reveals that antipsychotic polypharmacy was dispensed to 13.8% of people who were regularly dispensed an antipsychotic drug during 2007. The observed rate of use of antipsychotic polytherapy is similar to that reported in previous studies. For example, Kogut et al. (2005) [39] found, in a population drawn from US Medicaid users, that 10.1% were given antipsychotic polypharmacy. The criteria used by Kogut et al. to classify a patient as an antipsychotic user and to define antipsychotic polypharmacy were similar to those applied here. Also, other studies reported a prevalence of polypharmacy of 15.6% [17]. However, some studies have used an antipsychotic polypharmacy criterion that, in our view, is too broad (two or more antipsychotics used simultaneously) [16,40], thus yielding notably higher prescription rates (41% and 50%). Given that the central aim of the present study was to analyse the regular use of antipsychotic combinations, three restrictive criteria were applied to avoid including combinations when the patient was switching between antipsychotic treatments. Yang et al. (2008) [41], applying shorter time criteria (at least six weeks of simultaneous use and twelve weeks if one of the drugs used was clozapine), reported an increased rate of use from 1.9% in 1997 to 5.5% in 2000. Another study of community-treated patients with schizophrenia found that 42.5% were given more than one antipsychotic [42], although this variation may be due to differences in the methods used.

Overall, men were more likely to be given an antipsychotic when they were aged under 60. The profile of use of drug combinations in women extends beyond 80 years of age. The present findings are consistent with previous reports showing that antipsychotic polypharmacy is more widely used in men [39,42], possibly due to the increased severity of treatment-resistant schizophrenia among males [43].

The 57.8% of all combinations involved two antipsychotic drugs, most commonly oral risperidone plus quetiapine. Of those treated with combinations, 25.6% were given three antipsychotics. The 16.6% of patients were dispensed four or more antipsychotics.

One notable pattern of use identified in this study involved the combination of a FGA and a SGA (62.0%), which contrasts with previous reports in the literature (79.3% used two non-conventional antipsychotics or SGA) [39].

Clozapine was given to 1,615 (2.3%) patients, 41.3% of these in monotherapy and as part of antipsychotic polytherapy in 58.7%. The most common combination was clozapine plus amisulpride. The rate of clozapine use found in the present study is lower than that previously reported. For example, Pickar et al. (2008) [42] stated that 18% of their patients were given clozapine, although this higher rate may be related to the fact that they only included patients with severe schizophrenia treated in the community.

LAI formulations are frequently used to ensure treatment adherence that was not achieved with the oral formulation. Consequently, oral and LAI risperidone overlap could be accepted during a switching period from oral to LAI formulation, but not when used as a combination. As the criteria used to identify antipsychotic combinations tried to avoid the switching periods, the 162 patients identified in our study would correspond to combinations that are difficult to explain.

Clinicians continue to face numerous challenges in the treatment of schizophrenia, since ‘antipsychotic’ drugs do not function as ‘anti-schizophrenic’ drugs and do not act on comorbid conditions such as anxiety, or associated problems such as aggressiveness or suicidal risk. This leads to the use of multiple drug therapies, especially for reducing the mean length of hospital admissions. In our view, clinicians often base their arguments on the pharmacodynamic, pharmacokinetic or tolerance characteristics of different drugs, although psychiatrists generally consider antipsychotic polypharmacy as a largely ineffective strategy for patients who present resistant positive symptoms of psychosis [44].

Initiatives developed in this regard include the software package Psychiatric Clinical Knowledge Enhancement System (PSYCKES) [45], which enables clinicians to easily track the medication taken by their patients, as well as that prescribed by their peers, increasing the robustness of the decision-making process. A similar strategy was described in the Developing Evidence-Based Implementation Trial (DEBIT) [46], based on a multi-faceted intervention involving an educational/cognitive-behaviour therapy workbook, an educational visit to consultants and a reminder system on medication charts. Both strategies led to reductions in the costs resulting from polypharmacy, crucial when prescribing antipsychotic combinations.

### Limitations

The first limitation concerns the lack of diagnoses associated with the database used. Although antipsychotics are the drugs of choice for schizophrenia they may also be indicated in bipolar disorder, behavioural disorders associated with cognitive deterioration, and in other psychiatric or neurological disorders. It is important to clarify that the objective of the study is to describe the use of antipsychotic combination, independent of the diagnoses. Further research could be conducted linking our database with others containing diagnoses (since ours had no diagnoses). This could help to analyse the differences and preferences on the use of antipsychotic combinations depending on the diagnoses. Moreover, the lower rate of clozapine use found in the present study, compared with that reported in other studies, doesn’t necessarily mean a lower rate of use for schizophrenia, as this study includes patients with different possible diagnoses. The fact of not knowing the diagnosis makes it impossible to analyse the use of antipsychotic combinations depending on the severity of the illness. Also it’s impossible to know if there is a great potential for off label use in our sample.

The second limitation is that the analysis did not consider antipsychotic drugs prescribed neither in private practice nor to inpatients. The data correspond to drugs dispensed by pharmacies when presented with a prescription by a doctor from the Catalan National Health Service, which includes mental health centres, out-patient and emergency departments, community rehabilitation centres and other out-patient facilities. These account for the majority of prescriptions issued, since in our healthcare context the cost of these drugs makes it unlikely that they would be widely prescribed on a private basis. All the population from Catalonia is insured by the Catalan Health Service and pharmacy products are covered for the 98% of the patients insured. However, it is not possible to know precisely if we have considered all the antipsychotics used, due to the fact that part of the population may also have a private insurance and thus pay themselves for the treatment. Interventions that aim to promote the rational use of antipsychotics should also include prescription rates upon hospital discharge or in emergency units, since these can influence the maintenance treatment subsequently received by a patient.

The third limitation relays on the nature of our data. We used an administrative database of prescriptions processed by the National Health Service, and the measure taken into account was the number of packages dispensed to a patient upon presenting a prescription in a pharmacy. Therefore, the analysis concerned drugs dispensed rather than the prescription itself. It was also not possible to study the prescribed dose. Evaluating the treatment and its results, although interesting, was not an aim of our study. The prescribed dose could also affect the number of packages dispensed in a period of time, which would interfere with our study because the criteria we used to establish the study population were based on the number of packages dispensed. Similar studies using databases of medical records could complement the results of the present study.

Moreover, the criteria established to define the study sample tried to identify the simultaneous use of antipsychotic and distinguish it from the sequential use. The only way to accurately identify antipsychotic combination would have been through the prescription date but that was not available in our administrative database. Consequently, the filters used to identify antipsychotic combination were restrictive and a large proportion of patients were excluded from this definition and labelled as unspecified polytherapy.

The fourth limitation concerns the safety of using combinations of antipsychotics. As the data analysed do not include morbidity rates they are unable to shed any light on this aspect. In our view, there is a need for further research that takes an approach like the STAR*D [47] study with antidepressants, analysing different strategies of sequenced treatment with antipsychotics.

## Conclusions

Although antipsychotic polypharmacy is widely used in our healthcare setting, it appears not to follow the available clinical practice guidelines indicating the use of using antipsychotic monotherapy, not in combination. Although there is no evidence of any effective antipsychotic combination, it could be very interesting to analyse in depth which are the most appropriate antipsychotic combinations used in the population of Catalonia. This would guide psychiatrists and general practitioners in their everyday clinical practice. Developing strategies for enhancing the use of recommendations in clinical practice will render the work clinicians more efficient and understandable in the eyes of policy makers, and vice-versa.

## Competing interests

The authors declare that they have no competing interests.

## Authors’ contributions

All the authors of the article have participated in: 1) making substantial contributions to conception and design, or acquisition of data, or analysis and interpretation of data; 2) writing the drafts of the manuscript or revising them critically for important intellectual content; and 3) giving final approval of the version to be published.

## Pre-publication history

The pre-publication history for this paper can be accessed here:

http://www.biomedcentral.com/1471-244X/12/42/prepub
